# Comparing floral resource maps and land cover maps to predict predators and aphid suppression on field bean

**DOI:** 10.1007/s10980-021-01361-0

**Published:** 2021-11-19

**Authors:** Lolita Ammann, Aliette Bosem-Baillod, Philipp W. Eckerter, Martin H. Entling, Matthias Albrecht, Felix Herzog

**Affiliations:** 1 Agricultural Landscapes and Biodiversity, Agroscope, Reckenholzstrasse 191, 8046 Zürich, Switzerland; 2 Research Institute of Organic Agriculture (FiBL), Ackerstrasse 113, 5070 Frick, Switzerland; 3 iES Landau, University of Koblenz-Landau, Fortstrasse 7, 76829 Landau (Pfalz), Germany

**Keywords:** predation, pest control, landscape management, semi natural habitat, ecosystem services

## Abstract

**Context:**

Predatory insects contribute to the natural control of agricultural pests, but also use plant pollen or nectar as supplementary food resources. Resource maps have been proposed as an alternative to land cover maps for prediction of beneficial insects.

**Objectives:**

We aimed at predicting the abundance of crop pest predating insects and the pest control service they provide with both, detailed flower resource maps and land cover maps.

**Methods:**

We selected 19 landscapes of 500 m radius and mapped them with both approaches. In the centres of the landscapes, aphid predators – hoverflies (Diptera: Syrphidae), ladybeetles (Coleoptera: Coccinellidae) and lacewings (Neuroptera: Chrysopidae) – were surveyed in experimentally established faba bean phytometers (*Vicia faba* L. Var. Sutton Dwarf) and their control of introduced black bean aphids (*Aphis fabae* Scop.) was recorded.

**Results:**

Landscapes with higher proportions of forest edge as derived from land cover maps supported higher abundance of aphid predators, and high densities of aphid predators reduced aphid infestation on faba bean. Floral resource maps did not significantly predict predator abundance or aphid control services.

**Conclusions:**

Land cover maps allowed to relate landscape composition with predator abundance, showing positive effects of forest edges. Floral resource maps may have failed to better predict predators because other resources such as overwintering sites or alternative prey potentially play a more important role than floral resources. More research is needed to further improve our understanding of resource requirements beyond floral resource estimations and our understanding of their role for aphid predators at the landscape scale.

**Supplementary Information:**

The online version contains supplementary material available at 10.1007/s10980-021-01361-0.

## Introduction

As natural enemies of crop pests, pollinators and decomposers, insects provide important ecosystem services to agriculture (Losey and Vaughan 2006). Public awareness of declining insect numbers and risks associated with pesticide applications (EFSA 2015) increase the pressure on agriculture to find more sustainable management practices. The presence of predatory insects at the right moment and in sufficient quantity in agricultural fields can help to avoid insecticide applications against crop pests (Thies and Tscharntke 1999; Losey and Vaughan 2006; Tschumi et al. 2015). Thus, how to maintain and promote populations of natural enemies of crop pests in agroecosystems that spill into agricultural fields is of great interest. The conservation and restoration of areas of natural and semi-natural habitats (SNH) even in intensively used farmland is often (e.g. Tscharntke et al. 2012; Rusch et al. 2016; Sutter et al. 2018; Martin et al. 2019), but not always enhancing populations of predatory insects and the pest control services they provide (Tscharntke et al. 2016; Karp et al. 2018). A better understanding of which landscape and habitat features are critical for an effective conservation of predatory insects is therefore urgently needed. For example, SNH can not only host natural enemies, but also antagonists of natural enemies (Martin et al. 2013) or preferred hosts for pest species (Heimpel et al. 2010). SNH comprise a large set of different habitat types such as forest lots, hedgerows or grasslands (Herzog et al. 2017) that can differ significantly in their potential to sustain natural enemies (Schirmel et al. 2018; Bartual et al. 2019), providing food, shelter and overwintering sites (Fahrig et al. 2011; Holland et al. 2016). A better understanding of which features of such habitats drive predator numbers and thus the potential to contribute to natural pest control services would represent a big step towards more effective conservation biocontrol.

Many insect pest predators in agricultural landscapes rely on floral food resources to complete their life cycle (e.g. hoverflies, lacewings and parasitoids) or to overcome times of scarce prey supply (Landis et al. 2000; Symondson et al. 2002; Wäckers and Van Rijn 2012; Lu et al. 2014). For example, larval growth in ladybirds is enhanced by supplementary pollen supply and wild flower strips tailored to floral resource needs of predators efficiently enhances pest control in crops (Jonsson et al. 2015; Tschumi et al. 2015, 2016). Unlike wild flower strips, SNH such as forest edges are often located in some distance to the field and it is less clear, how their floral resources promote pest control in crops. To date, we lack knowledge on the response of predators to landscape scale floral resource availability based on flower availability in major habitat types including crops. By mapping and quantifying the availability of floral resource characteristics, we expected to gain important insights into predator requirements at landscape scale. Such refined “functional habitat maps” have been proposed to improve the prediction of species and functional groups (Vanreusel and Van Dyck 2007; Fahrig et al. 2011; Lausch et al. 2015) – although generating such maps is significantly more laborious than “classical” habitat mapping, where landscapes are characterized via coarse habitat classifications (e.g. cropping area and SNH; hereafter land cover maps). Knowing which floral resources predators require, and in which habitat types they prevail, will allow for specific recommendations on habitat and agricultural landscape design, provided that their population increase translates in improved pest control.

We asked the following research questions:

1. Do aphid predators increase with the amount of floral resources in the landscape?
2. Do aphid predators increase with the amount of SNH in the landscape?
3. Is there a habitat type of particular importance for floral resource availability, and for aphid predator abundances?
4. Do floral resource maps predict aphid predator abundance better than land cover maps?
5. Are black bean aphid populations reduced by predator numbers on faba bean?

## Methods

### Study design and experimental setup

**Fig. 1:**
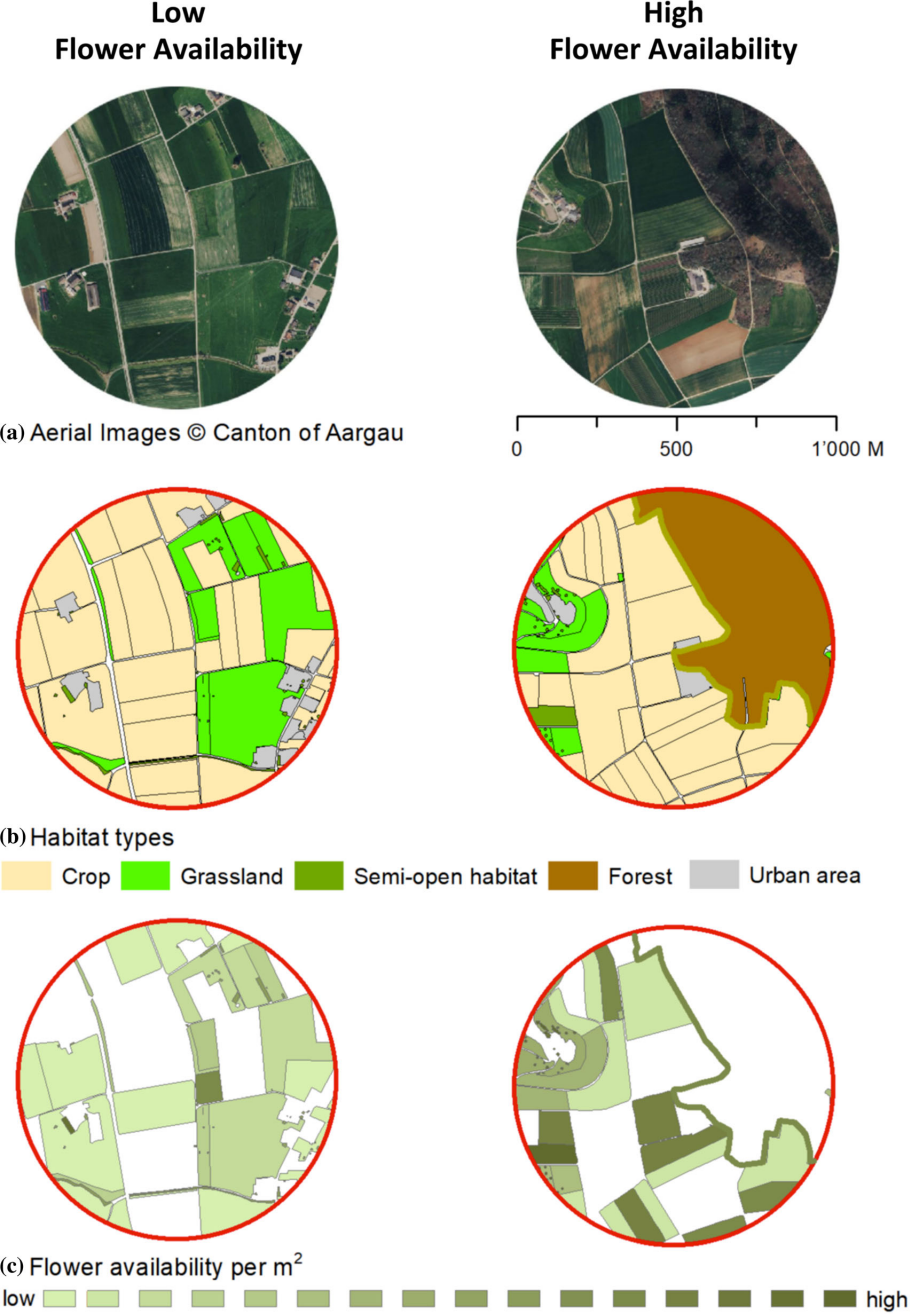
Two landscapes with high and low flower availability. Plot pair **a** shows the aerial image. Plot pair **b** shows habitat categories for land cover maps. Plot pair **c** shows average flower availability (m^3^ times flowering duration) per m^2^ for habitat sub-categories of the depicted landscapes (low = 0.0002, high = 1.3251)

A total of 19 agricultural landscape sectors of 500 m radius (hereafter landscapes) were selected in northern Switzerland, near Zürich (see supplementary material Fig. S1 for spatial distribution of landscapes). Landscapes covered a gradient of varying proportions of forest edges, semi-open habitats (hedgerows, tree rows and single trees), grasslands (intensively managed meadows, extensively managed meadows and pastures) and crops (mass-flowering crops, intensive orchards and ley meadows, i.e., non-permanent meadows as part of the crop rotation on arable land). Land cover maps of the four habitat types were established using aerial images that were verified and supplemented based on field observations (Fig. 1a and b) and amalgamated in ArcGIS (ESRI) with a minimal mapping unit of 1 sqm. Forest edges, semi-open habitats and grasslands were grouped as semi-natural habitats (SNH).

### Floral resource mapping

Floral resource maps were established according to the same four habitat categories as in land cover maps (forest edges, grasslands, semi-open habitat and crops). Floral resources were assessed in the field between beginning of April and mid-May 2017, the time period most relevant for the control of aphid pests in cereals, oilseed rape and fruit production of the study region (Stähler Pflanzenschutz, Switzerland). Flower availability was assessed as the total volume of open flowers in each landscape that was available to predators with exception of grasses. To account for differences in vegetation structure and composition between habitats, different methods were used for each habitat type. For grassland (i.e., permanent meadows and pastures), at least one large representative grassland patch of each management type (extensively managed meadow, intensively managed meadow, pasture) was selected in each landscape. In each of these grassland patches, flower density of all vascular flowering species were measured in 10 randomly located three-dimensional assessment cubes of 1 m^3^. To account for temporal variation in the floral composition and flower densities of flowering species, the measurement was repeated three times (every two to three weeks) during the sampling period. The volume of flower bearing plant parts in all woody species was estimated in the field with a 2 × 10 m ground resolution along every single woody element. To obtain species specific flower densities in tree crowns and shrubs, ten representative individuals per species were selected and their flower densities inside two 1m^3^ cubes per tree were determined. Flower densities in insect pollinated crops were assessed the same way with ten fields per crop type.

From these field measurements specific flower numbers within each landscape were calculated (flower density per m^3^ (*D*_species_) multiplied by flower bearing volume (*V*_species_) and either grassland area or crop area). To assess floral resource availability, species specific flower numbers were multiplied with the flower size (*S*_species_) and the flowering duration (*T*_species_). To determine flower availability (*F*_species_) on the landscape scale or within different habitat types, species specific flower availability was pooled either over landscapes or habitat types within landscapes. See supplementary material for a detailed description of the mapping procedures.

### Predator and aphid survey

In the center of each of the 19 landscapes, next to a winter wheat field, a patch of ten faba bean (*Vicia faba* L. Var. Sutton Dwarf) phytometer plants was established. Potted phytometer plants were exposed between wheat fields and adjacent grassy field margins as a highly standardized habitat. See supplementary material Fig. S2 for a graph of the experimental layout. Faba bean plants had been raised in an insect-proof greenhouse. At the start of bean flowering, 48 h before translocation to the field, plants were infested with approximately 20 black bean aphids (juvenile *Aphis fabae* Scop., purchased from Katz Biotech AG). Aphids were transferred on a single *V. faba* leaf, which was pinned below the uppermost crown of small leaves (i.e. at the youngest plant part) close to the stem. All black bean aphids were counted again immediately after translocation to the field (used as initial “starting population” number in the analyses) and on the last day of exposure, 14 days later. The numbers of predators (ladybirds, lacewings and hoverflies present as eggs, larvae, or adults on phytometer plants) were recorded after two days (approximately 48 h), four days (approx. 96 h) and 14 days after exposure. The few aphids that migrated from the environment into faba beans (e.g. *Megoura viciae* Buckton) were not taken into account for analysis, since numbers would rather relate to landscape scale aphid pools than predation on the faba beans. Furthermore, aphid mummies of parasitoids were counted as regular black bean aphids as mummification could not be reliably determined after less than two weeks of development (often inflated appearance, but no change in colour yet).

### Statistical analysis

*Aphis fabae* population growth (as an estimate of aphid control, with high growth rates reflecting low aphid control and low growth rates reflecting high aphid control) was defined as the difference in population size between the first day of exposure and the last day of exposure (14 days later). For both days, *Aphis fabae* numbers were pooled over all bean phytometer plants within a landscape. Numbers of ladybeetles, hoverflies and lacewings per landscape were pooled across the insect life stages, sampling rounds and individual phytometer plants per landscape. Pooling was necessary to minimize potential issues due to relatively low numbers in some of the observed predator taxa and thus to improve model fit and robustness. Relations between predators and aphid control, as well as their relation to landscape parameters, were assessed with linear regression models. For each response variable (predators, aphid control) and each map type (classical habitat maps and functional resource maps based on flower availability) a separate model was computed to avoid correlation of variables and overfitting by backwards model selection (Dormann et al., 2018). Each model included the four habitat categories as predictors (grasslands + semi-open + forest edges + crops). To test effects from SNH, habitat areas of semi-open habitat, grasslands and forest edges were pooled and tested against predators and aphid control in separate models. Landscape level flower availability was derived from pooling over habitat types and tested separately, as for SNH (see Table 1). To address the relatively high content in zeroes of the predator response and the data structure given by predator progeny, a two step modelling approach (Cunningham and Lindenmayer 2005) was chosen for predators on the model structure as described above. In a first step, binomial models were fitted for a predator presence and absence response, while in the second step linear regression models were performed excluding landscapes with zero counts. This approach yielded quantitatively the same results as a simple linear regression approach or a permutation test (500 permutations). Generalized R-squared values were generated for a measure of goodness of fit (Zhang 2017). To meet linear model assumptions (normality, homoscedasticity), predator numbers were log-transformed. Potential collinearity between explanatory variables was checked based on variation inflation factors (VIFs; car package version 3.0-2; Fox 2018), making sure a threshold of three was not reached (Zuur et al. 2007). All analysis were performed using R version 3.4.1 (Team 2017).

**Table 1:** Results of linear regression models of landscape variables and aphid predator numbers (ladybirds, lacewings, hoverflies; log_10_-transformed) on aphid control (reduction in aphid population growth during field exposure) on faba bean. With a two-step modelling approach accounting for zero-inflation in aphid predator response: Binomial models on presence-absence response of predators to landscape variables (step one) and linear regression models on log-transformed aphid predators (zero counts excluded) in response to landscape variables (step two)Significant p-values are indicated in bold (p < 0.05). See material and methods section for detailed information on models and parameters SNH Semi-natural habitat (grassland, forest edge, semi-open habitat)with aResults of two-step modelling approach accounting for zero-inflation in aphid predator response

Response	Fixed effect	Model	df	AICc	Gen. R^2^	Habitat	Std. Coeff.	*F*-value	*p*-value
Predators	Habitat area	Step 1	17	21.86	0.115	SNH	−2.225	2.434	0.137
		Step 2	13	28.38	0.002	SNH	−0.042	0.024	0.880
	Total flower abundance	Step 1	17	23.44	0.034	Landscape-level	−1.292	0.788	0.387
		Step 2	13	26.74	0.059	Landscape-level	0.244	0.821	0.381
	**Habitat area**	**Step 1**	**14**	**25.54**	**0.412**	Crop	3.264	0.909	0.357
						Grassland	−2.055	0.957	0.345
						**Forest edge**	**7.104**	**4.999**	**0.042**
						Semi-open	−4.112	3.377	0.087
		**Step 2**	**10**	**34.88**	**0.408**	Crop	0.504	1.883	0.200
						Grassland	0.027	0.009	0.925
						**Forest edge**	**0.764**	**6.400**	**0.030**
						Semi-open	−0.127	0.133	0.723
	Flower abundance	Step 1	14	27.95	0.279	Crop	−1.455	0.529	0.479
						Grassland	−1.089	0.465	0.506
						Forest edge	2.169	1.266	0.279
						Semi-open	−3.233	3.461	0.084
		Step 2	10	39.67	0.182	Crop	0.232	0.631	0.445
						Grassland	0.234	0.490	0.500
						Forest edge	0.307	0.926	0.359
						Semi-open	−0.324	0.806	0.391
Aphid control	Habitat area		17	329.51	0.123	SNH	0.351	2.383	0.141
	Total flower abundance		17	331.94	0.001	Landscape-level	−0.057	0.055	0.817
	Habitat area		14	339.13	0.201	Crop	−0.272	0.639	0.438
						Grassland	0.299	1.097	0.313
						Forest edge	−0.275	0.910	0.356
						Semi-open	0.048	0.021	0.888
	Flower abundance		14	339.34	0.192	Crop	−0.023	0.009	0.926
						Grassland	0.427	2.448	0.140
						Forest edge	−0.146	0.356	0.560
						Semi-open	−0.040	0.021	0.886
	**Predators**		**17**	**326.70**	**0.261**	–	**−5.211**	**5.993**	**0.026**

## Results

Crops covered on average around 30% of the landscape, providing more than 50% of floral resources available (56% provided by *Brassica napus*), but only 12% of floral diversity (Fig. S3). Grasslands and forest edges provided the highest amounts of flower diversity (33% and 31% respectively) but contributed relatively little to total flower availability (2%, of which 47% were *Taraxacum officinale*, and 14%, of which 20% were *Prunus spp*., respectively). Grasslands covered 12% of the landscape while forest edges covered a very small proportion (<5%). Semi-open habitat (traditional orchards), provided almost 30% of total flower availability which was less diverse than forest edges (22% of total landscape-level diversity). However, the two woody SNH (forest edges and semi-open habitat) provided by far the highest diversity as well as the highest flower availability relative to the area covered (Fig. S3d, e; supplementary material, Table S2).

**Fig. 2:**
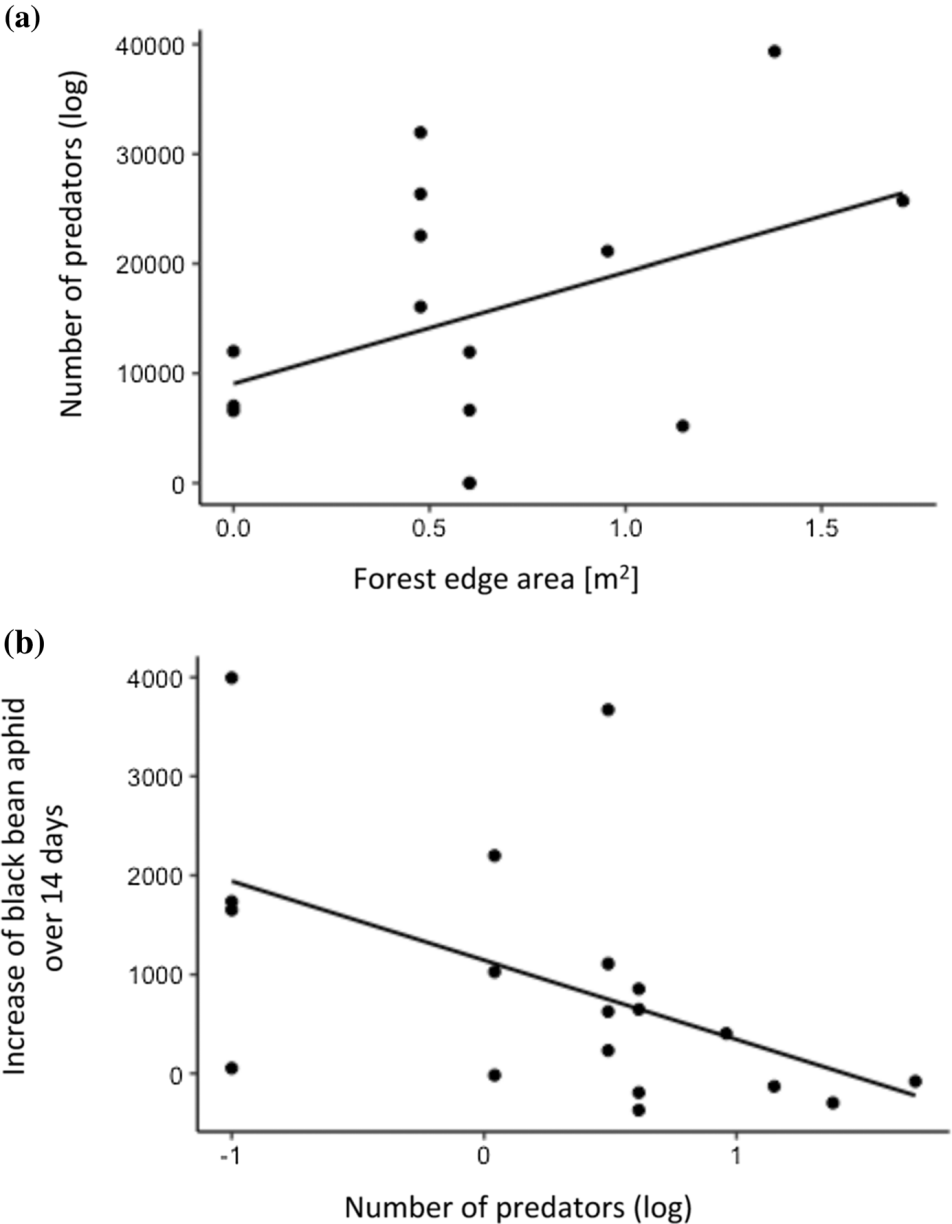
Number of predators on faba beans (ladybirds, lacewings and hoverflies on 10 phytometer plants per landscape) in relation to **a** amount of forest edge habitat in the landscape and **b** aphid control (restriction of black bean aphid population growth over 14 days on faba bean) (see Table 1 for parameters)

A total of 129 predators were recorded on the bean phytometer plants, of which 63% were ladybirds, 28% hoverflies and 9% lacewings. SNH area covered more than 10% of the total landscape area but did not significantly explain predator numbers or aphid control. However, when separating habitat categories into finer components (forest edges, crop, grasslands, semi-open habitat), predator numbers increased with the proportion of forest edge (Table 1; Fig. 2). No other habitat type could explain predators significantly, neither from functional resource maps, nor from land cover maps. Thus, functional resource maps did not improve prediction over land cover maps.

Across all landscapes, the average number of black bean aphids on field bean phytometer plants increased from 283.2 (± 26.3) per site after translocation of invested plants to 1183.8 (± 289.8) two weeks later. Aphid population growth was suppressed in landscapes with higher numbers of aphid predators I.e. aphid control was positively related to predator numbers (Table 1; Fig. 2) but did not relate to any landscape descriptor that predators were tested for (Table 1).

## Discussion

In the present study, numbers of studied aphid predators on faba beans, i.e., hoverflies, lady beetles and lacewings, could be explained with land cover maps, but not with floral resource maps, although they consume floral resources at least in certain life stages or to supplement their prey diet. Thus, predictions of e.g. Dennis et al. (2006), Moore et al. (2010) or Turlure et al. (2019) that resource maps or “functional habitat maps” would better predict the occurrence of target organisms for biodiversity conservation and ecosystem service promotion than land cover maps (based on land use or vegetation types) could not be confirmed for the case of aphid predators and floral resources maps. Potential reasons for this finding are discussed in the following. This study also highlights the importance of forest edges in agricultural landscapes to enhance aphid predators on crop plants.

Natural enemies and pest control have been shown to be related to landscape-level environmental traits (Bianchi et al. 2006; Chaplin-Kramer et al. 2011; Veres et al. 2013; Rusch et al. 2016) but such relationships are often system specific while it remains challenging to draw general conclusions about their response to non-crop semi-natural habitats (Karp et al. 2018). These inconsistent responses may be partly related to structural differences between studied SNH types. Our results show how variable different types of SHN are at least regarding their floral resource provisioning. Failure of floral resource maps to predict aphid predators on faba bean may suggest the need for further improvement of floral resource maps; grouping of floral resources according the commonly used land cover types allows direct comparison of land cover maps and floral resource maps. However, different classifications of floral resources might be more in accordance with predators’ needs (Fahrig 2011). Results may also show the need for functional resource maps based on alternative drivers, such as prey availability, overwintering habitat or shelter to predict predator abundance (Landis et al. 2000; Sarthou et al. 2005; Burgio et al. 2006; Schirmel et al. 2018). Findings in this and previous studies indicate that forest edges may be particularly important in providing such resources (e.g. Sarthou et al. 2005). Consequently, several studies in different agricultural systems have found positive relationships between forest habitats and predator abundance, as well as pest control services (Nicholls et al. 2001; Alhmedi et al. 2009; Gardiner et al. 2009; Mitchell et al. 2014), despite the fact that such habitats may also promote crop pests (e.g. Kheirodin 2020). In our study, an increasing proportion of forest edge habitat was related to higher predator abundance, which in turn was positively associated with aphid control (Fig. 2). Forest edges are considered as an important habitat for many natural enemies (Ingrao et al. 2017; Schirmel et al. 2018; Bartual et al. 2019) in terms of facilitated movement of predators between habitats and as an important source of prey and shelter (reviewed by Holland et al. 2016). For example, stinging nettles (*Urtica dioica*), prevalently found along forest edges in the studied landscapes, are hosts of important alternative ladybird prey (Ammann et al. 2020) and were found to host ladybirds as well as hoverflies prior to crop colonisation (Alhmedi et al. 2009).

On the other hand, a review by Holland et al. (2016) found grassy habitats to be at least equally important for the predicting predators, which contrasts with our findings. Grasslands, similar to crops, differ in their management and the associated degree of disturbance experienced by predators (Giller 1997), a factor not investigated in this study. The short time periods during which floral resources are provided by crops may be a further reason for the lack of prediction of predators by crops (Schellhorn et al. 2015; Baude et al. 2016).

Whether floral resources are an important limiting factor and therefore improve prediction of flower-visiting insects compared to land cover maps seems to depend on factors such as the agricultural system investigated, and the group of flower-visiting insects assessed. Pollinators, such as bees, which exclusively rely on floral resources in contrast to the aphid predators studied here, should show closer relationships to floral resources than aphid predators that consume floral resources only in certain life stages or as complementary food resources in addition to animal preys (e.g. Williams et al. 2012; Bertrand et al. 2019; Albrecht et al. 2020; Eckerter et al. 2020). In fact, also Bartual et al. (2019) did not detect floral resource effects on predators on a landscape scale, while wild bee abundance could be significantly better predicted if floral resources were considered in addition to land cover categories.

To our knowledge this is one of the first studies evaluating flower resources with this degree of detail at landscape level, and relating these floral resources to crop pest predators while at the same time analyzing pest predator abundance in relation to pest control (Vialatte 2017). This laborious mapping of flower resources at the landscape scale revealed that more than 50% of landscape-level floral resources during the studied time period were provided by crops, of which the large majority came from oilseed rape and fruit trees. However, such crops have relatively short flowering periods and provide abundant floral resources only during short time periods in contrast to grasslands, which provide constant floral supply but 25 times less floral resources in total. Woody habitats provide high floral resource availability per area.

Floral resource mapping at the landscape is challenging and time consuming. To minimize inaccuracies, data collection in the field was whenever possible restricted to counting, measuring and presence-absence characterization of landscape parameters, avoiding observer bias through estimates. However, estimating floral resources at the landscape scale inevitably relies on some generalizations resulting in potential over- or underestimations in the contribution of different flowering species or habitats to the landscape scale floral resource availability. Even though we assessed floral resources in the time period most relevant for pest control of most crops grown in the study region (beginning of April to mid-May), we cannot exclude the possibility that floral resources outside this time period would better predict predators. Peaks of high pollen availability offered by grasslands later in the season, could potentially be important, especially for long lived or multivoltine natural enemies (Fiedler and Landis 2007). Moreover, floral resource availability during the previous season could play a role in predicting natural enemy abundance in crops in the following year. Although floral resource availability likely varies to some extent across years, at least floral resources provided by the permanent habitat types studied is likely to be similar across subsequent years. Considering the rarely achieved sampling effort and the fact that the same methodology was consistently applied across all landscapes, we are confident that our floral resource maps are of high quality and allow for robust comparisons across landscapes. Nevertheless, mapping could be further improved by more precise monitoring of flowering durations, mapping of forest interiors, and monitoring of floral resources over entire activity periods of predators.

## Conclusions

Against our expectations, floral resource maps performed poorly at predicting the studied flower-visiting aphid predators. Land cover maps allowed to explain aphid predators better than floral resource maps. For a deeper understanding of predator requirements to the landscape we either require more suitable floral resource maps, or functional resource maps based on resources other than flowers. Still, our findings indicate that the broad dichotomous classifications of habitat types into SNH and crop habitat, sometimes termed “landscape structure” and “matrix”, is not sufficient. Instead, different types of SNH (and possibly crops, depending on the purpose of the investigation) should be differentiated. This differentiation revealed an important role of forest edges in promoting aphid predators on faba beans, which should be considered in conservation biological control management in the studied agricultural landscapes. Besides floral resource provisioning, other resources (i.e., shelter, overwintering opportunities, alternative prey) offered by forest edges and other SNH should be taken into account to promote predators of crop pests and the service they provide.

## Supplementary Information

Below is the link to the electronic supplementary material.

Supplementary material 1 (PDF 9706 kb)

## Data Availability

The datasets generated during and/or analysed during the current study are available from the corresponding author on reasonable request.

## Code Availability

No substantial code was necessary for this study.
