# Supplementary material for: Comparing floral resource maps and land cover maps to predict predators and aphid suppression on field bean
Source: Landsc Ecol. 2021 Nov 19;37(2):431–41. doi: 10.1007/s10980-021-01361-0 (PMC8841323; doi:10.1007/s10980-021-01361-0)
Supplement: Supplementary file 1 — Supplementary material 1 (PDF 9706 kb) [file 10980_2021_1361_MOESM1_ESM.pdf]

Comparing floral resource maps and land cover maps to predict predators and aphid suppression on field bean

Lolita Ammann[1], Aliette Bosem-Baillod[2], Philipp W. Eckerter[3], Martin H. Entling[3], Matthias Albrecht[1], Felix Herzog[1]

[1]*Agroscope, Agricultural Landscapes and Biodiversity, Reckenholzstrasse 191, 8046 Zürich, Switzerland;* [2]*Research Institute of Organic Agriculture FiBL, Ackerstrasse 113, CH-5070 Frick, Switzerland;* [3]*University of Koblenz-Landau, iES Landau, Fortstrasse 7, 76829 Landau (Pfalz), Germany*

Corresponding author:
Lolita Ammann
email: lolo.ammann@gmail.com

Supplementary information on landscape mappings

General approach

Nineteen landscape sectors of 500 radius were selected along a gradient of varying shares of forest edges, semi-open habitats (hedgerows, tree rows and single trees), grasslands (permanent intensively managed meadows, permanent extensively managed meadows and pastures) and crops (mass-flowering crops, intensive orchards and ley meadows (i.e., non-permanent meadows as part of the crop rotation on arable land) (Fig. 1). Landscapes were selected to form a gradient in habitat proportion of the four habitat types that did not exceed variation inflation factors (VIF; Fox 2018) of more than 3 (Zuur et al. 2007).

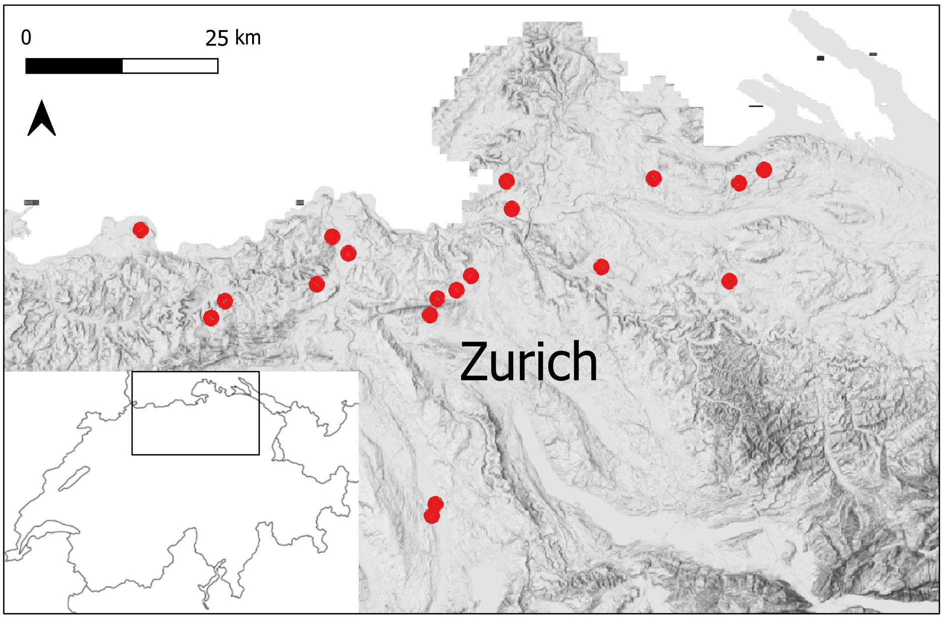

Fig. S1 Geographical distribution of landscapes in northern Switzerland (Map type: swissALTI3D relief shading, source: Federal office of topography, swisstopo)

Predictive performance of land cover maps was compared to functional resource maps that held information on flower availability and on flower diversity within each of the four habitat types. Floral resources were assessed in the field between the beginning of April and mid-May 2017. Flower availability assessments were done according to floral composition and structure of different habitat

types. For example, along all linear woody elements, the flower bearing crown volume of woody species was mapped comprehensively. Floral availability was then calculated from crown volumes and species specific floral traits (flower size, flower density, flower duration) retained from individual representatives of the species. Floral resources in grasslands were assessed in each landscape separately. In each landscape one representative grassland was chosen per management type (e.g. extensively mangaged, intensively managed) and representative flower densities of all flowering species were counted in ten times 1 m^3 within each chosen grassland. The procedure for meadows was repeated three times, to account for temporal variation in floral composition. This appendix describes in detail, how flower availability and diversity were assessed in the four habitat types.

Calculation of flower availability

Flower availability was assessed as the total volume of open flowers in each landscape and habitat that was available to predators except for grasses. To account for differences in vegetation structure and composition between habitats, different methods were used for each habitat type. For each grassland (i.e., permanent meadows and pastures), at least one large representative patch of each management type (extensively managed meadow, intensively managed meadow, pasture) was selected in each landscape. In each of these grassland patches, flower density of all vascular flowering species (except grasses) were measured in 10 randomly located three-dimensional assessment cubes of 1 m^3. To account for temporal variation in the floral composition and flower densities of flowering species, the measurement was repeated three times (every two to three weeks) during the sampling period. The resulting level of replication per landscape (n = 19) varied between 1-5 meadows multiplied by ten cubes per meadow and three sampling rounds. The volume of flower bearing plant parts in all woody species was estimated in the field with a 2x10 m ground resolution along every single woody element. To obtain species specific flower densities in tree crowns and shrubs, ten representative individuals per species were selected and their flower densities inside two 1 m^3 cubes per tree were determined. Flower densities in insect pollinated crops were assessed the same way with ten fields per crop type.
From these field measurements species specific flower numbers within each landscape were calculated (flower density per m^3 (D_{species}) multiplied by flower bearing volume of woody plants (V_{species}), grassland area or crop area respectively). To assess floral resource availability, species specific flower numbers were multiplied with the flower size (S_{species}) and the flowering duration (T_{species}). To determine flower availability (F_{species}) on the landscape scale or within different habitat types, species specific flower availability was pooled either over landscapes or habitat types within landscapes.

$$F_{\text{species}} = S_{\text{species}} \times D_{\text{species}} \times V_{\text{species}} \times T_{\text{species}}$$

$$F_{\text{Landscape}} = \sum_{species} F_{\text{species}}$$

Flower diversity was calculated using the Simpson index (Simpson 1949; implemented in the R vegan package 2.5-2 (Oksanen et al., 2018)), based on flower availability per habitat type. Mapping resulted in two types of floral resource maps: flower availability and flower diversity (Fig. 1c, 1d).

Species' flower volume (S_{species})

Numbers of flowers will not necessarily translate directly into floral resource availability to insects. Depending on size and floral traits, the amount and accessibility of nectar and pollen varies. We therefore explored the relationships of flower area (projection area from the top) and flower volume (approximated as cylinders using flower diameter as cylinder width and corolla depth as cylinder length) with floral nectar availability of 72 plant species frequently flowering in the study region using

the extensive database provided by Baude et al. (2016). We also explored the relationship of these flower traits with pollen volumes provided for flowers of 27 flowering plant species (Hicks et al. 2016). Flower diameter and corolla depth were obtained from a floral trait database compiled for most flowering plant species of the study region Frey et al. (*in prep*). For most floral types, flower volumes were taken from individual flowers, except for Asteraceae (inflorescence diameter used as cylinder width) and male catkin flowers, since recognising open flowers was difficult. For species lacking information in the trait database, values were obtained from own measurements of flowers in the study region, or average values of other species of the same genus represented in the trait database used. If values varied strongly among species of the same genus, the value of the most similar species with a similar geographical distribution was used (according to Info Flora; Juillerat et al. 2017). Flower volume (log-transformed) showed close and significant positive linear relationships in regression models with nectar ($df = 1$, $t = 3.42$, $P = 0.001$) and pollen availability ($df = 1$, $t = 12.04$, $P < 0.001$).

Floral resource accessibility

Many predators lack the long tongues of bees that would allow them to access most flowers. Therefore, they usually rely on relatively simple floral shapes, with open access to pollen and shallow corolla tubes for access of nectar (Colley and Luna 2000; Fiedler and Landis 2007; Haaland et al. 2011). Flowers with no open access to pollen or nectar were excluded from analysis. Nectar access was categorised as open, if pollinator behaviour recorded by Frey et al. (*in prep*) was classified as primitive, nectar tube length was shorter than 1 mm and van Rijn and Wäckers 2016 did not note else based on experimental data. Pollen access was categorised as open if Frey et al. (*in prep*) classified the flower associated pollinator behaviour either as primitive or for crawling in. Pollen access was categorised as not possible if pollen resources were marked as hidden or buzzing pollinators were needed. Since most flowers had open pollen access, results did not deviate from analysis without this pre-selection.

Flower density ($D_{species}$)

Flower density describes the number of open flowers in the flowering parts of the species during its flowering period. Flower density was assessed differently for woody plants, arable crops and grasslands. Flower density in woody species (trees and shrubs of forest edges, hedgerows, orchards and single trees) was assessed by counting the number of flowers in flowering parts of trees and shrubs within 20 cubes of 1 m^3 (two cubes each in 10 representatives per species) during the species' flowering period.

Flower density in crops (including lay grasslands) was based on counts in 10 cubes of 1 x 1 x 1 m size in two fields per crop type during the peak flowering period.

Flower density assessments in grasslands were more complicated. Grasslands vary in flower composition depending on management, season and factors like soil types and exposition, which leads to differences between landscapes. For this reason, grasslands were classified into three management types: Permanent meadows and extensively managed meadows and pastures. Flower densities of grassland species were assessed per grassland type and landscape from beginning of April until mid-May, in roughly three week intervals. Flower densities per species in grasslands were counted in 10 x 1 m^3 cubes in 2 representatives (if present) per grassland type (20 cubes) per landscape (19) per sampling round (3).

Mapping of woody plants to estimate $V_{species}$ of flowering tree and shrub species

To quantify potentially flower bearing plant volumes in the landscape, approaches optimised for the different habitat types were applied. For crops und grasslands this was done by transferring square meters retained from areal maps into cubic meters, since none of them have flower horizons higher

than one meter. To estimate the floral resource contribution of tree and shrub species along forest edges and hedgerows in a landscape, the volume of flowering parts of all tree and shrub species potentially visited by insects for floral resource use were estimated along the entire length of all forest edges and hedgerows in each landscape (ca. 38 km). To this end, forest edges and hedgerows were split into segments of two meters (covering the entire width of the woody vegetation of hedgerows, and a depth of ten meters into the forest along forest edges). Within each segment, the presence of all woody species was recorded and the volumes of the flowering parts of each species in the upper crown layer, the middle crown layer, and the shrub layer were estimated. Volumes of flower parts in hedgerows and of the middle crown layer and the shrub layer of forest edges were directly estimated in the field. Since estimates on high trees are difficult and become un-precise, a GIS approach using a digital vegetation height map was applied to assess the height of the upper crown layer to estimate flower part volumes of this layer: woody elements were digitized in ArcGIS version 10.6. (ESRI) and a vegetation height map with a 1 m resolution available for Switzerland (Ginzler 2018) was placed over the orthophoto. To avoid underestimation of vegetation height along forest edges with relatively sparse tree cover, average maximum tree height per segment was used. The height of the upper crown layer was calculated by subtracting the height minus the height of the upper part of the middle crown layer recorded in the field. Ground-truthing confirmed that this approach yielded reasonably precise and robust estimations of tree heights and estimates of flowering crown volumes of the upper crown layer.

Isolated trees identified on the orthophoto were assigned to species in the field and the outline digitised as polygon in ArcGIS. Volumes of flowering trees were approximated based on estimated crown projection area and crown height. Crown volumes of fruit trees in intensive orchards were calculated by multiplying orchard area with estimated ratio of tree coverage and a standard approximation of 2 m crown height. Crown volumes of fruit trees in high-stem traditional fruit orchards were calculated the same way but with a standard approximation of 5 m crown height (Anbautechnik Bioobst, FiBL).

Estimation of flowering period ($T_{species}$)

For flowering trees, shrubs and crops average flowering duration was set to 21 days based on observations in the field. Flowering season started 10 days prior to the recorded flowering peak and ended 10 days after the flowering peak. For grasslands it was possible to determine the flowering period based on continuous floral assessments during the season: a species' flowering period was defined as the period from the first to the last day it was recorded flowering in a sample plot. Very rare flowering grassland species that occurred in less than 1 % of all sampling plots (22 species) were excluded from further analyses. Due to the rare occurrence they did not allow to retain reliable flowering duration.

Flower diversity

Flower diversity defined by the Simson index (Simpson 1949; implemented in the R vegan package 2.5-2 (Oksanen et al. 2018)) was calculated from species specific flower availability of each habitat type (grassland, crop, semi-open or forest edge) and landscape.

Experimental setup for survey of natural enemies and aphids

In the center of each landscape, at the edge of a winter wheat field, a patch of ten faba bean (*Vicia faba* L. Var. Sutton Dwarf) phytometer plants was established.

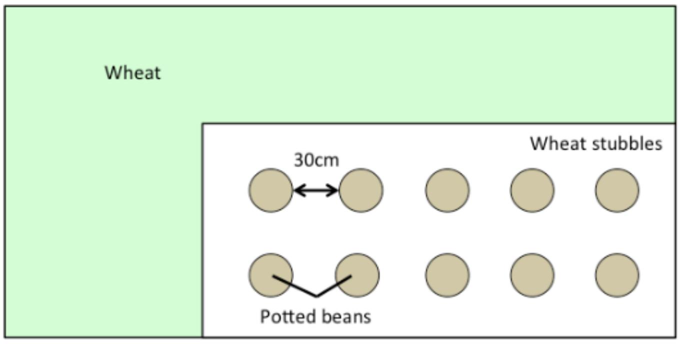

Fig. S2 Faba bean setup in wheat fields

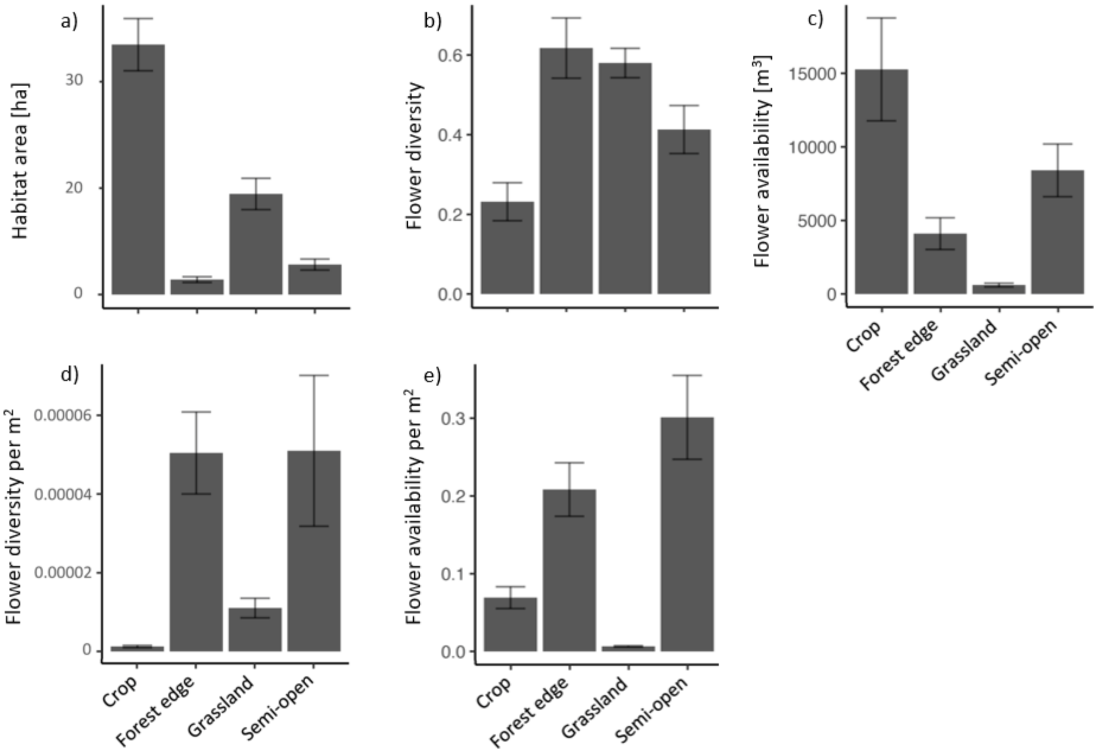

Fig. S3 Distribution of a) average habitat area, b) average flower diversity (Simpson's index), c) average flower availability (flower volume * flowering days), d) average flower diversity per habitat area (Simpson's index) and e) average flower availability per habitat area over landscapes for the four habitat types (+/- standard error). See Appendix for detailed information on calculation of flower abundance and diversity.

Table S1. Results of linear regression models of flower diversity and aphid predator numbers (ladybirds, lacewings, hoverflies; log_{10}-transformed) on aphid control (reduction in aphid population growth during field

exposure) on faba bean. Results of two-step modeling approach accounting for zero-inflation in aphid predator response: Quasi-binomial models on presence-absence response of predators to flower diversity (step one) and linear regression models on log-transformed aphid predators (zero counts excluded) in response to flower diversity (step two). Significant *p*-values are indicated in bold ($p < 0.05$).

Response	Fixed effect	Model	df	AICc	Habitat	Std. Coeff.	*F*-value	*p*-value
Predators	Total flower diversity	Step 1	17	24.31	Landscape-level	-0.057	0.001	0.970
		Step 2	13	27.80	Landscape-level	-0.199	0.537	0.477
Aphid control	Flower diversity	Step 1	14	31.31	Crop	-0.604	0.077	0.786
					Grassland	0.609	0.092	0.766
					Forest edge	2.489	1.897	0.190
					Semi-open	-0.110	0.004	0.951
		Step 2	10	37.52	Crop	-0.372	1.519	0.246
					Grassland	0.348	1.226	0.294
					Forest edge	0.167	0.325	0.581
					Semi-open	-0.497	2.973	0.115
	**Predators**		**17**	**29.70**	**-**	**5.211**	**5.211**	**0.036**

Table S2. Flowering plants recorded from April to mid-May 2017 in four different habitat types. Habitat types include: Forest edges, semi-open habitats (hedgerows, tree rows and single trees), grasslands (intensively

managed meadows, extensively managed meadows and pastures) and crops (mass-flowering crops, intensive orchards and ley meadows, i.e., non-permanent meadows as part of the crop rotation on arable land).

**Habitat category**	**Taxon**
Semi-open habitat	Abies alba
Semi-open habitat	Acer campestre
Semi-open habitat	Acer platanoides
Semi-open habitat	Acer pseudoplatanus
Semi-open habitat	Acer sp.
Semi-open habitat	Aesculus hippocastanum
Semi-open habitat	Betula sp.
Semi-open habitat	Carpinus betulus
Semi-open habitat	Crataegus monogyna
Semi-open habitat	Euonymus europaeus
Semi-open habitat	Fagus sylvatica
Semi-open habitat	Forsythia intermedia
Semi-open habitat	Fraxinus excelsior
Semi-open habitat	Juglans regia
Semi-open habitat	Larix decidua
Semi-open habitat	Lonicera xylosteum
Semi-open habitat	Malus sp. cultivated
Semi-open habitat	Picea abies
Semi-open habitat	Pinus sylvestris
Semi-open habitat	Prunus avium wild
Semi-open habitat	Prunus padus
Semi-open habitat	Prunus sp.
Semi-open habitat	Prunus sp. cultivated
Semi-open habitat	Prunus spinosa
Semi-open habitat	Pyrus sp. cultivated
Semi-open habitat	Quercus sp.
Semi-open habitat	Robinia pseudoacacia
Semi-open habitat	Salix sp.
Semi-open habitat	Sorbus sp.
Semi-open habitat	Tilia sp.
Semi-open habitat	Viburnum lantana
Semi-open habitat	Viburnum opulus
Grassland	Ajuga reptans
Grassland	Anthriscus sylvestris
Grassland	Bellis perennis
Grassland	Capsella bursa-pastoris
Grassland	Cardamine hirsuta
Grassland	Cardamine pratensis
Grassland	Cerastium sp.
Grassland	Crepis biennis
Grassland	Daucus carota
Grassland	Fragaria vesca
Grassland	Galium sp.
Grassland	Geranium sp.
Grassland	Glechoma hederacea
Grassland	Heracleum sphondylium
Grassland	Knautia arvensis
Grassland	Lamium purpureum
Grassland	Leucanthemum vulgare
Grassland	Lotus corniculatus
Grassland	Medicago lupulina
Grassland	Myosotis arvensis
Grassland	Onobrychis viciifolia
Grassland	Plantago sp.

Grassland	Potentilla reptans
Grassland	Primula sp.
Grassland	Ranunculus sp.
Grassland	Rhinanthus alectorolophus
Grassland	Rumex acetosa
Grassland	Salvia pratensis
Grassland	Sanguisorba minor
Grassland	Silene dioica
Grassland	Silene pratensis
Grassland	Stellaria media
Grassland	Taraxacum officinale
Grassland	Tragopogon pratensis
Grassland	Trifolium campestre
Grassland	Trifolium dubium
Grassland	Trifolium incarnatum
Grassland	Trifolium pratense
Grassland	Trifolium repens
Grassland	Valerianella locusta
Grassland	Veronica sp.
Grassland	Vicia sepium
Grassland	Viola reichenbachiana
Forest edge	Abies alba
Forest edge	Acer campestre
Forest edge	Acer platanoides
Forest edge	Acer pseudoplatanus
Forest edge	Acer sp.
Forest edge	Aesculus hippocastanum
Forest edge	Betula sp.
Forest edge	Carpinus betulus
Forest edge	Crataegus monogyna
Forest edge	Euonymus europaeus
Forest edge	Fagus sylvatica
Forest edge	Fraxinus excelsior
Forest edge	Juglans regia
Forest edge	Larix decidua
Forest edge	Lonicera xylosteum
Forest edge	Malus domestica
Forest edge	Picea abies
Forest edge	Pinus sylvestris
Forest edge	Prunus avium wild
Forest edge	Prunus padus
Forest edge	Prunus sp.
Forest edge	Prunus spinosa
Forest edge	Quercus sp.
Forest edge	Robinia pseudoacacia
Forest edge	Rubus sp.
Forest edge	Salix sp.
Forest edge	Sorbus sp.
Forest edge	Viburnum lantana
Forest edge	Viburnum opulus
Crop	Ajuga reptans
Crop	Bellis perennis
Crop	Brassica napus
Crop	Capsella bursa-pastoris
Crop	Cardamine hirsuta
Crop	Cardamine pratensis
Crop	Galium sp.

Crop	Lamium purpureum
Crop	Malus sp. cultivated
Crop	Medicago lupulina
Crop	Myosotis arvensis
Crop	Plantago sp.
Crop	Prunus sp. cultivated
Crop	Pyrus sp. cultivated
Crop	Ranunculus sp.
Crop	Rumex acetosa
Crop	Stellaria media
Crop	Taraxacum officinale
Crop	Trifolium incarnatum
Crop	Trifolium pratense
Crop	Trifolium repens

Bibliography

Baude M, Kunin WE, Boatman ND, et al (2016) Historical nectar assessment reveals the fall and rise

of floral resources in Britain. Nature 530:85
Colley MR, Luna JM (2000) Relative Attractiveness of Potential Beneficial Insectary Plants to Aphidophagous Hoverflies (Diptera: Syrphidae). 1054–1059
Fiedler AK, Landis DA (2007) Plant characteristics associated with natural enemy abundance at Michigan native plants. Environ Entomol 36:878–886
Fox J (2018) CRAN task view: Statistics for the social sciences
Frey D, Amman L, Albrecht M, Moretti M (*in prep.*) Functional and structural blossom and flower traits of animal pollinated plants of urban gardens.
Haaland C, Naisbit RE, Bersier LF (2011) Sown wildflower strips for insect conservation: A review. Insect Conserv. Divers.
Hicks DM, Ouvrard P, Baldock KCR, et al (2016) Food for Pollinators : Quantifying the Nectar and Pollen Resources of Urban Flower Meadows. 1–37. https://doi.org/10.1371/journal.pone.0158117
Juillerat P, Bäumler B, Bornand C, et al (2017) Flora Helvetica Checklist 2017: der Gefässpflanzen der Schweiz= de la flore vasculaire de la Suisse= della flora vascolare della Svizzera
Oksanen J, Blanchet FG, Friendly M, et al (2018) vegan: Community Ecology Package. R package version 2.5-2. 2018
Simpson EH (1949) Measurement of diversity. Nature 163:688
van Rijn PCJ, Wäckers FL (2016) Nectar accessibility determines fitness, flower choice and abundance of hoverflies that provide natural pest control. J Appl Ecol 53:925–933. https://doi.org/10.1111/1365-2664.12605
Zuur A, Ieno EN, Smith GM (2007) Analyzing ecological data. Springer
